# Influence of a high-altitude hypoxic environment on human plasma microRNA profiles

**DOI:** 10.1038/srep15156

**Published:** 2015-10-15

**Authors:** Yan Yan, Yonghui Shi, Cheng Wang, Pengtao Guo, Junjun Wang, Chen-Yu Zhang, Chunni Zhang

**Affiliations:** 1 Department of Clinical Laboratory, Jinling Hospital, State Key Laboratory of Analytical Chemistry for Life Science, Nanjing University School of Medicine, Nanjing University, Nanjing 210002, China; 2 Jiangsu Engineering Research Center for microRNA Biology and Biotechnology, Advance Research Institute of Life Sciences, Nanjing University, Nanjing, China; 3 Department of Clinical Laboratory, the Forty-First Hospital of PLA, Nêdong, China

## Abstract

Circulating microRNAs (miRNAs) are promising disease biomarkers. However, the influence of high-altitude hypoxic environments on plasma miRNA profiles remains unknown. This study included a total of 509 plasma samples from 278 native Tibetans and 80 newly arrived migrant Han Chinese (Tibet Han) residing at 3560 m and 151 Han Chinese residing at 8.9 m (Nanjing Han). The levels of 754 miRNAs were initially determined using a TaqMan Low Density Array (TLDA) in two pooled samples from 50 Tibet Han and 50 Nanjing Han individuals. Some markedly altered miRNAs in Tibet Han were subsequently measured in all 509 plasma samples by individual qRT-PCR. Compared with the Nanjing Han, 172 miRNAs were differentially expressed in the Tibet Han (105 upregulated and 67 downregulated). The correlation coefficient for the two groups was 0.72. Several upregulated miRNAs were randomly selected for analysis by qRT-PCR, and the results were consistent with those identified by TLDA. These miRNAs were also significantly increased in the Tibetans compared with the Nanjing Han. Furthermore, these altered miRNAs showed strong positive correlations with red blood cell counts and hemoglobin values. These data are the first to provide clear evidence that a high-altitude hypoxic environment significantly affects human plasma miRNA profiles.

MicroRNAs (miRNAs) are a class of small non-coding RNAs of ~22 nucleotides in length that regulate gene expression at the posttranscriptional level by either degrading or blocking the translation of messenger RNA targets. The involvement of miRNAs in a variety of physiological and pathological conditions has been demonstrated^1^,^2^. Recent studies by our group and others have shown that circulating miRNAs are stably detectable and may serve as potential biomarkers for cancer and non-cancer diseases^3^,^4^,^5^,^6^,^7^. Therefore, circulating miRNA expression profiles are being intensively investigated for their involvement in various pathogenic processes. To translate miRNA assays from the bench side to the bedside, the pre-assay conditions of human circulating miRNA profiles must be determined. Circulating miRNA concentrations can be affected by some confounding factors, such as age and sex^8^. However, the influence of environmental factors, such as living conditions, residence and altitude, have not been determined.

Among the most severe environmental challenges to confront human is the reduced oxygen availability in high-altitude regions, such as in the Tibetan Plateau^9^. The Tibetan Plateau is the highest plateau in the world with an average altitude of over 4500 m, and it is inhospitable to human settlement because of cold temperatures and low atmospheric oxygen pressure (~40% lower than at sea level)^10^. When lowlanders immigrate to high-altitude locations, they are not well adapted to the high-altitude hypoxic conditions. For most individuals, acclimatization to low oxygen involves a marked increase in hemoglobin (HGB) levels^10^. Recently, a few reports revealed that hypoxia was an important proximal regulator of miRNA biogenesis and function and that miRNAs could rapidly respond to the stress caused by hypoxia by post-transcriptional translational modulation at the cellular level^11^,^12^,^13^. Therefore, it is reasonable to speculate that high-altitude hypoxic environments are likely to have an impact on circulating miRNA concentrations.

To test our hypothesis, in the present study, we performed high-throughput TaqMan Low Density Array (TLDA) screening followed by confirmation using a quantitative reverse-transcription polymerase chain reaction (qRT-PCR) assay to systematically and comprehensively evaluate and compare plasma miRNA profiles between Han Chinese residing in Nanjing at sea level (Nanjing Han) and Han Chinese recently immigrated from the plains to Tibet (Tibet Han) as well as ethnic Tibetans. We also analysed the correlations between the altered plasma miRNAs and hematological indices.

## Results

### TLDA chip analysis of plasma miRNA profiles

**Figure 1:**
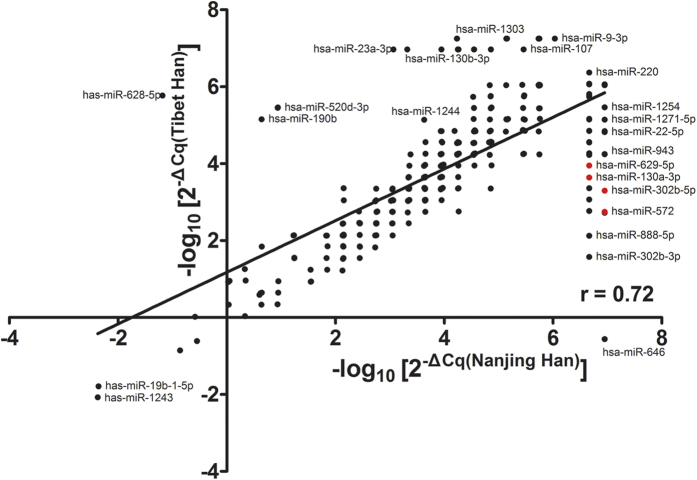
Pearson correlation scatter plot of plasma miRNA levels in the Tibet Han and Nanjing Han groups as determined by TLDA.

First, we globally analysed the plasma miRNA expression profiles from the Tibet Han and Nanjing Han samples using a TLDA chip. Fifty plasma samples randomly selected from 80 Tibet Han people and 151 Nanjing Han people were separately pooled to form two sample pools for the TLDA analysis. The mean age and sex distribution of the 50 Nanjing Han were exactly matched with those of the 50 Tibet Han (34.49 ± 9.17 years, 28 male/22 female *vs*. 35.38 ± 11.69 years, 32 male/18 female; *P* = 0.765 and *P* = 0.414, respectively). Pearson correlation analysis showed the correlation coefficient (r) for the two groups to be 0.72 (Fig. 1). The miRNAs assessed were considered to be upregulated if their quantification cycle (Cq) values were <35 in the Tibet Han sample and downregulated if their Cq values were <35 in the Nanjing Han sample and if there was a 2-fold difference in the expression level between the two groups. Of the 754 miRNAs tested, 172 miRNAs were differently expressed in the following manner: 105 miRNAs were upregulated and 67 miRNAs downregulated in the Tibet Han group compared with the Nanjing Han group (Supplementary Table S1). Forty-five of the markedly altered miRNAs from the Tibet Han group (change fold >100) are listed in Table 1. The TLDA results demonstrated that the plasma miRNA patterns in the Tibet Han group were markedly different from those in the Nanjing Han group.

We used the miRBase database (version 22.0) to analyse whether the markedly upregulated or downregulated miRNAs in Table 1 are part of families residing in the same genomic location. miRNA gene clusters were named based on their close physical distances to genes on the same chromosome (<10 kb). We found that miR-495 and miR-323a-3p, miR-500a-5p and miR-501-5p, miR-518e-3p and miR-520d-3p, and miR-516a-3p and miR-522-3p are each a set of two members of four miRNA clusters, and each pair is likely to be co-regulated.

### Confirmation of the TLDA results using individual qRT-PCR assays

Next, to confirm the TLDA results, we carried out qRT-PCR assays of miRNAs with individual plasma samples. Four plasma miRNAs that were upregulated in the Tibet Han group compared with the Nanjing Han group upon TLDA analysis (Fig. 1 and Supplementary Table S1); specifically, miR-130a-3p, miR-302b-5p, miR-572 and miR-629-5p, were randomly selected for qRT-PCR validation with individual plasma samples from 80 Tibet Han and 151 Nanjing Han persons. In addition, these 4 miRNAs were also examined in the plasma samples from 278 ethnic Tibetan highlanders. Table 2 summarizes the demographic and hematological features of all participants. No significant differences in age or sex were identified among the Tibet Han, Nanjing Han and Tibetan groups. However, the red blood cell counts (RBC), HGB, hematocrit (HCT) and mean corpuscular hemoglobin (MCH) values of both males and females were significantly increased (*P* < 0.005), whereas blood platelet (PLT) levels were markedly decreased (*P* < 0.001) in the Tibet Han group compared with the Nanjing Han group. The HGB, HCT and MCH values of both males and females, along with the female RBC values, were also significantly increased in the Tibetan group compared with the Nanjing Han group, whereas PLT levels were markedly decreased between the two groups (*P* < 0.001); however, no marked difference in male RBC values was observed between the two groups. In addition, the male RBC, HGB and HCT values were significantly increased in the Tibet Han group compared with those of the Tibetan group (*P* < 0.001).

**Figure 2:**
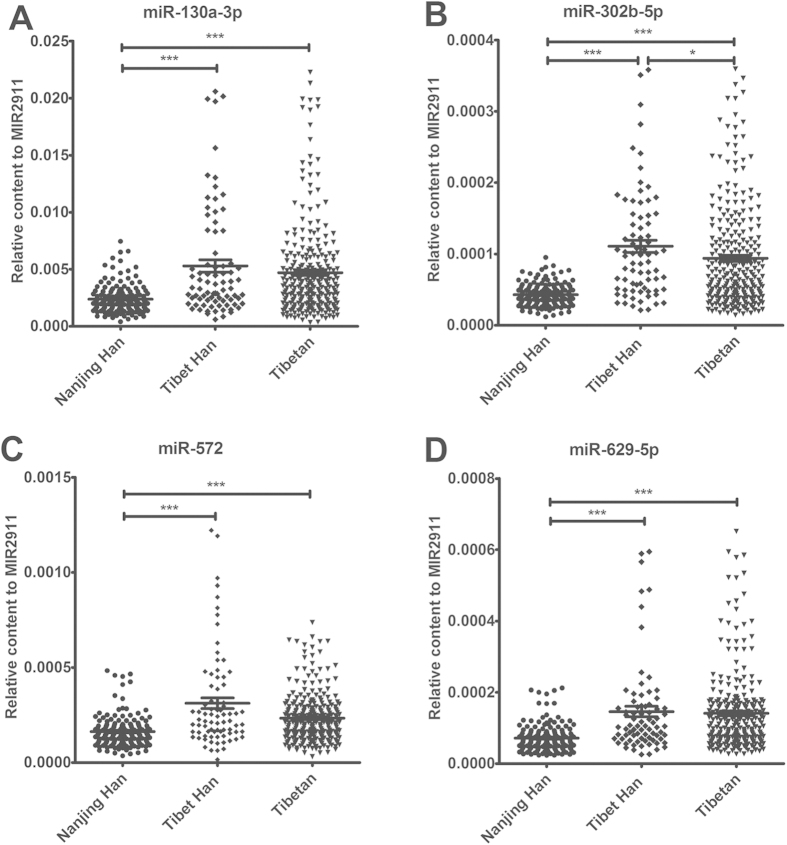
The relative concentrations of miR-130a-3p, miR-302b-5p, miR-572 and miR-629-5p in the plasma samples from the Nanjing Han (n = 151), Tibet Han (n = 80) and Tibetan (n = 278) groups (A–D). Cq values were converted to relative concentrations normalized to MIR2911 values and were calculated using the comparative Cq method (2^−ΔCq^). Each point represents the mean of triplicate samples. ^*^P < 0.05; ^***^P < 0.0001.

The results of the individual qRT-PCR assays were in agreement with those of the TLDA analysis of the pooled samples showing that the plasma concentrations of all 4 of the selected miRNAs were significantly increased in the Tibet Han group compared with the Nanjing Han group (fold change >2.0 and *P* < 0.0001) (Fig. 2 and Supplementary Table S2). Furthermore, the levels of the 4 miRNAs were also markedly higher in the Tibetan group than in the Nanjing Han group (*P* < 0.0001) (Fig. 2 and Supplementary Table S2). However, the Tibet Han and Tibetan groups were not significantly different with respect to the concentrations of the 4 miRNAs with the exception of miR-302b-5p, which was higher in the Tibet Han group than in the Tibetan group (*P* = 0.016).

### Expression levels of the validated upregulated miRNAs in peripheral blood cells

We measured the levels of the 4 validated upregulated miRNAs in peripheral blood cell samples of 30 Nanjing Han, 30 Tibet Han and 30 Tibetan individuals. The results showed that the expression levels of these 4 miRNAs were significantly higher in the Tibet Han and Tibetan groups than in the Nanjing Han group (*P* < 0.05), whereas no marked difference in these miRNAs was observed between the Tibet Han and Tibetan groups (Supplementary Fig. S3).

### The co-correlation of the validated hypoxia miRNAs and their relationships with hematological indices

We used a Spearman rank correlation analysis to test for the co-correlation between the 4 validated increased plasma miRNAs in all studied individuals. As shown in Supplementary Fig. S4, a strong positive correlation was observed between the 4 miRNAs (*P* < 0.0001). These results indicate that some individuals have higher miRNA responses overall. Furthermore, we evaluated whether these 4 miRNAs were related to hematological parameters. We used Spearman rank correlation analysis to test for correlations between each of these 4 miRNAs and the measured hematological parameters. All 4 miRNAs showed strong positive correlations with RBC, HGB and HCT (*P* < 0.01) and significantly negative relationships with PLT (*P* < 0.05) (Table 3 and Supplementary Fig. S5–Fig. S7). Together with the qRT-PCR results, these data suggest that the upregulation of these 4 miRNAs in populations residing in Tibet may be a result of the high-altitude hypoxic environment.

### Target analysis of miRNAs altered by high-altitude hypoxia

To explore the possible roles and molecular basis of the high-altitude-altered miRNAs in response to a hypoxic environment, we performed a bioinformatics prediction of the potential target genes associated with hypoxia through the use of several widely utilized target prediction databases, including TargetScan, miRanda and PicTar. Computational analysis revealed that some hypoxia-related genes, such as DDX6, hypoxia-inducible factors-3 (HIF-3), vascular endothelial growth factor-A (VEGFA) and egl nine homologue 1 (EGLN1), were potential target genes of the validated miRNAs (Table 4). In addition, several erythroid-related and megakaryocytic-related genes, such as SMAD5 and ETV6, were also predicted to be potential targets of miR-130a-3p and miR-302b-5p (Table 4).

Subsequently, we measured the plasma concentrations of the growth factors VEGFA and erythropoietin (EPO) in the plasma samples from 64 Nanjing Han, 46 Tibet Han and 64 Tibetan individuals using an enzyme-linked immunosorbent assay (ELISA). The plasma concentrations of both VEGFA and EPO were found to be significantly elevated in the Tibet Han (*P* < 0.05 and *P* < 0.001, respectively) and Tibetan groups (*P* = 0.001 and *P* < 0.001, respectively) compared with the Nanjing Han group; however, no marked difference in the two growth factors was observed between the Tibet Han and Tibetan groups (*P* > 0.05) (Supplementary Table S3). Furthermore, we evaluated the associations between VEGFA, EPO and the expression levels of the 4 validated hypoxia miRNAs using Spearman rank correlation analysis. The plasma concentrations of both VEGFA and EPO exhibited strong positive correlations with the expression levels of the 4 miRNAs (Supplementary Fig. S8 and Fig. S9).

## Discussion

miRNAs have been increasingly recognized as biomarkers for diverse classes of disease; however, the influences of pre-assay conditions, such as the effects of environmental factors on human circulating miRNA profiles, are largely unknown. In this study, we investigated for the first time the influence of a high-altitude hypoxic environment on human plasma miRNA profiles. By performing a genome-wide plasma miRNA differentiation scan using a TLDA chip followed by qRT-PCR validation, we demonstrated that the plasma miRNA profile of Han Chinese who had recently immigrated to highland Tibet is dramatically different from that of those residing at sea level. More importantly, the upregulated miRNAs showed strong positive correlations with RBC, HGB and HCT values. These results suggest that a high-altitude hypoxic environment has a remarkable influence on human plasma miRNA patterns.

The main physiological challenge in high-altitude plateau environments is hypoxia. When people living in a plain environment migrate to a plateau, they face the threat of hypoxia^14^. Physiological responses to high-altitude hypoxia are complex and involve a range of mechanisms^15^. Previous studies have discovered a special group of miRNAs that are differentially expressed in response to hypoxia, and altered miRNA concentration profiles, or signatures, from various organisms, cell types, and disease states in relation to hypoxia have been determined^16^,^17^,^18^. However, these studies mainly focused on miRNA expression levels in tissues or cells. Considering that miRNAs are key elements in the response to hypoxia, we speculated that high-altitude hypoxic environments are likely to have an impact on human circulating miRNA patterns. To test our hypothesis, we used a high-throughput TLDA assay to comparatively analyse miRNA expression profiles of the Tibet Han and Nanjing Han populations. As expected, the expression profiles of plasma miRNAs from the Tibet Han were dramatically different from those of the Nanjing Han, with 172 differently expressed miRNAs. Next, we used qRT-PCR assays to confirm the TLDA results. We randomly selected 4 miRNAs to measure their concentrations by qRT-PCR in individual plasma samples in a large validation cohort. Consequently, the results of the individual qRT-PCR assays were in accordance with those of the TLDA analysis showing that the plasma concentrations of these selected miRNAs were significantly increased in the Tibet Han group compared with the Nanjing Han group. To confirm that the miRNA dysregulation observed in the Tibet Han was the result of a high-altitude hypoxic environment, we also examined these markedly altered miRNAs in native Tibetan highlanders. We found that the levels of all 4 miRNAs were also markedly higher in the Tibetan highlanders than in the Han lowlanders, whereas most of these miRNAs did not show any significant difference between the native Tibetan and Tibet Han individuals. Furthermore, we observed a similar trend of these miRNAs in the peripheral blood cells of the studied individuals, suggesting that these plasma miRNA levels may reflect cellular changes in the populations investigated. These results demonstrate that a number of miRNAs were differentially expressed in the circulation in response to a high-altitude hypoxic environment. In addition, these hypoxia-associated miRNAs exhibited strong positive correlations, indicating that some individuals have a higher overall miRNA response when exposed to high altitude.

Hypoxia, or low oxygen availability, has a complex and extensive impact on human and animal physiology, and elaborate adaptive mechanisms have evolved to respond to hypoxic stress. Of the 4 high-altitude hypoxia-upregulated plasma miRNAs identified in our study, miR-130a-3p and miR-572 have been reported to be upregulated in cells under hypoxic conditions^19^,^20^. In addition, a subset of the altered miRNAs (e.g., miR-21, miR-24, miR-30b, miR-224) identified during our TLDA analysis has also been associated with the hypoxic response in mammalian cells by other investigators^17^,^21^. These miRNAs may be regarded as general responders to hypoxia. Functional studies of miRNAs that respond during hypoxia may be helpful in uncovering the molecular basis of hypoxic acclimatization and illuminating the complexity of the hypoxia-response pathways in humans. Interestingly, by analysing the resulting data sets based on multiple target prediction algorithms, we found that some target genes of our identified miRNAs are involved in oxygen sensing and metabolism. For instance, DDX6, a DEAD-box helicase, was predicted to be the target gene of miR-130a. Decreased expression of DDX6 by miR-130a has been found to enhance the translation of hypoxia-inducible factor-1alpha (HIF-1α), a key transcription factor in the cellular response to hypoxia, in an internal ribosome entry site^19^. HIF-3, another oxygen-dependent transcription factor that activates a distinct transcriptional response to hypoxia^22^, was predicted to be a target gene of miR-629-5p. In addition, some genes, including EGLN1 and peroxisome proliferator-activated receptor alpha (PPARA), which are involved in the HIF pathway, were potential target genes of miR-302b-5p. EGLN1 plays a central role in the activation of hypoxia-inducible genes and the homeostasis of HIF under hypoxia and normoxia^23^,^24^. EGLN1 targets two HIFα proteins for degradation under normoxic conditions, decreasing the transcription of HIF-regulated targets, such as EPO, the erythropoietin gene whose product induces RBC production^9^. PPARA is also associated with HIF activity. PPARA expression is repressed by HIF1 during hypoxia^9^,^25^, and genes targeted by HIF are regulated by a HIF-independent mechanism involving PPARG coactivator-1α^26^.

In our study, we found that the plasma concentrations of the growth factors VEGFA and EPO exhibited strong positive correlations with the expression levels of the validated hypoxia-associated miRNAs. VEGFA was predicted to be a potential target of miR-302b-5p by bioinformatics analysis. The exact reason for the positive relationship between miR-302b-5p and VEGFA is currently unknown. Nevertheless, a recent study has demonstrated that hypoxia increases VEGFA expression in a number of epithelial cells^27^. Interestingly, our results also revealed that hypoxia could induce miR-302b-5p expression in peripheral blood cells. This evidence indicates that elevated VEGFA and miR-302b-5p levels may originate from different cells under hypoxic conditions and can explain why they show a strong positive correlation even though VEGFA is a potential target of miR-302b-5p. However, further studies are necessary both to clarify this issue and to validate the target genes of these miRNAs and the mechanisms by which circulating miRNAs respond to hypoxia.

In this study, we also tested the association of the validated upregulated miRNAs in the Tibet Han with several hematological indices that had distinctively high levels in the newly arrived migrant individuals originating from low-altitude populations. The specialized function of red blood cells is to transport O_2_ from pulmonary capillaries to tissue capillaries, where it is exchanged for CO_2_. When lowlanders immigrate to high altitude locations, they are not well adapted to the high-altitude hypoxic conditions, and for most individuals, acclimatization to low oxygen involves rapid increases in blood RBC, HGB and HCT levels^9^,^10^,^28^. In our study, we observed that RBC, HGB and HCT levels were significantly higher in both the Tibet Han and Tibetan groups than in the lowlander Han group, and these hematological indices were higher in the Tibet Han group than in the Tibetan group, which was consistent with previous reports^29^,^30^. It is worth mentioning that, in addition to these hematological indices, the levels of miR-302b-5p, a high-altitude hypoxia-upregulated miRNA validated in our study, was also higher in the Tibet Han than in the native Tibet population. The reason for this phenomenon may be that the physiological response to low oxygen of individuals of low-altitude origin is different than that of native Tibetans highlanders. Tibetans have lived at very high altitudes for thousands of years, and they have evolved a blunted physiological response to high-altitude hypoxia^9^. Furthermore, we used Spearman rank correlation analysis to test for correlations between the 4 plasma miRNAs and the hematological indices. We found that all 4 of the miRNAs showed strong and significant positive correlations with RBC, HGB and HCT values. These data provide evidence for the expression/activation of an array of circulating miRNAs in a highly coordinated manner with other physiological responses, such as increased hematological responses, to achieve enhanced cell survival under hypoxic conditions. In addition, target prediction revealed that some genes with important roles in erythroid and megakaryocytic differentiation were potential targets of our identified miRNAs. For example, genes related to the erythropoietin, such as SMAD5^31^ and SOX6^32^, and transcription factors with well-known functions in megakaryocytopoiesis, such as MAFB^33^ and ETV6^34^, are putative targets for miR-130a-3p and miR-302b-5p, respectively. These results indicate that miR-130a-3p and miR-302b-5p could be involved in regulating erythroid and megakaryocytic differentiation and are likely involved in high-altitude acclimatization and/or adaptation. Further study regarding the alteration and the roles of circulating miRNAs in physiological responses may shed light on the connections between these miRNAs and altitude acclamation.

In a previous study, it was observed that with increasing altitude, men had progressively more HGB than women in the Han but not the Tibetan population, indicating that a gender-related difference was greater in Han than in Tibetan individuals. Consequently, the difference in HGB values between the Tibet Han men and Tibetan men was larger than the Tibet Han women and Tibetan women^29^. A similar phenomenon was found in our study. The reasons underlying the progressively higher HGB in Han men compared with Han women as altitude increases are unknown. One possible reason, we suspect, is that Han men may have a stronger chronic erythropoietic response to altitude than their female counterparts. Further study is needed to clarify this issue.

In conclusion, we have demonstrated for the first time that a high-altitude hypoxic environment has a marked effect on human plasma miRNA expression profiles. In particular, we have identified a set of high-altitude hypoxia-altered plasma miRNAs that show strong positive correlations with hematological phenotypes. These data provide new insight into the physiological traits related to hypoxia and the molecular mechanisms involved in the hypoxic response, which has important implications for the prevention and treatment of some hypoxia-related diseases, such as mountain sickness and high-altitude pulmonary disease.

## Materials and Methods

### Participants and plasma sample processing

The study population consisted of a total of 509 plasma samples from 80 ethnic Han Chinese who immigrated one to two years ago (mean 17 months) from the plains (Jiangsu Province, Anhui Province and Shanghai City of China) to Nêdong County, a county of Lhoka prefecture in the Tibet Autonomous Region with an altitude of 3560 m (Tibet Han); 151 Han Chinese residing in Nanjing (Nanjing Han), a city of Jiangsu Province, with an altitude of 8.9 m; and 278 native Tibetans residing in Nêdong since birth. The Tibet Han and Tibetan study participants were recruited from individuals seeking routine health check-ups at the Forty-First Hospital of PLA in Nêdong, China, and those in the Nanjing Han group were recruited from individuals who had visited the Jinling Hospital, Nanjing, China, for routine check-ups between 2013 and 2014. Jiangsu, Anhui and Shanghai are all in the east of China and close to each other. The genetic backgrounds of the Tibet Han were matched with the Nanjing Han^35^.

All blood samples were collected in EDTA tubes using a standard operating procedure. All blood donors participating in this study provided written informed consent, and the ethics committees of the Forty-First Hospital of PLA and Jinling Hospital approved the study protocol in accordance with the Declaration of Helsinki.

### RNA isolation, TLDA and qRT-PCR

We carried out RNA isolation and measurements as previously described^5^. In brief, blood was centrifuged at 3000 rpm for 10 min, and the plasma supernatant was then collected and stored at −80 °C until RNA extraction. For TLDA, RNA was extracted using TRIzol reagent (Invitrogen, Carlsbad, CA, USA) from two pooled samples from 50 Tibet Han people or 50 Nanjing Han people (each plasma sample was 250 μL, and each pool contained 12.5 mL). The TLDA was carried out on an ABI PRISM 7900HT Sequence Detection System (Applied Biosystems, Foster City, CA, USA) to analyse the miRNA profiles of 754 different human miRNAs. The concentrations of the miRNAs are presented as Cq values and were normalized to an internal reference gene recommended by the manufacturer. The relative miRNA concentrations were calculated by the comparative Cq method (2^−ΔCq^). For the qRT-PCR assay, the total RNA was extracted from 100 μL individual plasma samples using a 1-step phenol/chloroform purification protocol. A TaqMan probe-based qRT-PCR assay was conducted on a 7300 Sequence Detection System (Applied Biosystems). All reactions were run in triplicate, and the average Cq values were calculated. To control for variability in the RNA extraction and purification procedures, an exogenous reference gene, plant miRNA MIR2911 (5′-GGCCGGGGGACGGGCUGGGA), was spiked into each sample at a final concentration of 10^6^ fmol/L during RNA isolation. This gene served as a synthetic external reference for the normalization of plasma miRNAs as it has no mammalian homologue and the measurement of its expression has high repeatability and reproducibility (Supplementary Methods and Supplementary Fig. S1 and Fig. S2). The relative levels of miRNAs were normalized to MIR2911 and were calculated using the comparative Cq method (2^−ΔCq^). The total RNA of peripheral blood cells was extracted with TRIzol Reagent according to the manufacturer’s instructions. The concentration and quality of the extracted RNA was determined using a spectrophotometer (Eppendorf, Hamburg, Germany) at 260 nm and 280 nm. To calculate the relative expression levels of target miRNAs, U6 was used as an internal reference for the qRT-PCR analysis of miRNAs in peripheral blood cells.

### Hematology and growth factor analysis

Hematological indices, including RBC, HGB, HCT, MCH and PLT levels, were measured using XE-2100 analysers (Sysmex, Kobe, Japan) with commercial reagents immediately after blood collection. Growth factors including EPO and VEGFA were measured using commercial ELISA kits (Cusabio, Wuhan, China).

### Statistical analysis

All statistical analyses were performed using the Statistical Analysis System software SPSS 17.0. Data are presented as the mean ± SEM for miRNAs or the mean ± s.d. for other variables. The differences in variants among groups were analysed using a one-way ANOVA, and the differences between groups were subsequently determined using a nonparametric Mann-Whitney U-test. A two-sided x^2^ test was used to compare sex distribution between two groups. A *P*-value < 0.05 was considered statistically significant. Correlations of miRNA profiles between two groups were calculated by using Pearson correlation analysis, and the co-correlation of the miRNAs and their relationship with other parameters were calculated using Spearman rank correlation analysis.

## Additional Information

**How to cite this article**: Yan, Y. *et al*. Influence of a high-altitude hypoxic environment on human plasma microRNA profiles. *Sci. Rep*. **5**, 15156; doi: 10.1038/srep15156 (2015).

## Supplementary Material

Supplementary Information

## Figures and Tables

**Table 1:** Markedly altered miRNAs in pooled plasma samples from the Tibet Han group compared with those from the Nanjing Han group as determined by TLDA.

miRNA	Nanjing Han	Tibet Han	Fold change (Tibet Han/Nanjing Han)
	ΔCq	ΔCq	
up-regulated			
miR-646	23.084635	−1.8663454	32433477.92
miR-302b-3p	22.145533	5.234892	123199.8522
miR-888-5p	22.145533	7.0637035	34680.31783
miR-661	23.084635	9.050674	16774.25416
miR-572	23.084635	9.1250515	15931.37867
miR-22-3p	22.145533	9.203108	7871.511633
miR-302b-5p	23.084635	10.959819	4466.150028
let-7a-5p	22.145533	10.19775	3950.399928
miR-501-5p	22.145533	11.163097	2023.217913
miR-130a-3p	22.145533	12.085655	1067.394672
miR-629-5p	22.145533	13.130329	517.4243003
miR-943	23.084635	14.099673	506.6908619
miR-106b-3p	23.084635	14.117106	500.6050380
miR-326	22.145533	14.051384	273.2635074
miR-500a-5p	22.145533	14.100896	264.0444487
miR-148b-3p	22.145533	14.130302	258.7169920
miR-511	22.145533	14.1374855	257.4319843
miR-517a-3p	22.145533	14.186428	248.8452433
miR-22-5p	23.084635	16.014692	134.358427
miR-548d-5p	22.145533	15.116016	130.6458058
miR-363-3p	22.145533	15.142132	128.3021023
miR-323a-3p	22.145533	15.148914	127.7003793
miR-1282	23.084635	16.088182	127.6856866
miR-598	22.145533	15.151174	127.5004917
miR-495	22.145533	15.151276	127.4914776
miR-638	23.084635	16.094122	127.1610479
miR-590-5p	22.145533	15.156548	127.0264392
down-regulated			
miR-628-5p	−3.9325724	19.176819	1.10505E-07
miR-523-3p	3.1135654	18.137585	3.00137E-05
miR-190b	2.1210022	17.119005	3.05599E-05
miR-520d-3p	3.111105	18.108130	3.05806E-05
miR-23a-3p	10.182589	23.153664	0.000124542
miR-130b-3p	11.027242	23.153664	0.000223657
miR-296-3p	13.109695	23.153664	0.000947249
miR-1303	14.054570	24.090704	0.000952407
miR-605	14.062584	24.090704	0.000957712
miR-124-3p	14.112743	23.153664	0.001898504
miR-503	14.135433	23.153664	0.001928599
miR-512-3p	14.135928	23.153664	0.001929261
miR-219-5p	14.161407	23.153664	0.001963636
miR-576-3p	15.103367	23.153664	0.003772412
miR-522-3p	15.112633	23.153664	0.003796719
miR-518e-3p	16.134354	23.153664	0.007708629
miR-516a-3p	17.075134	24.090704	0.007728638
miR-15a-3p	17.118477	24.090704	0.007964354

**Table 2:** Demographic and hematological features of the Nanjing Han, Tibet Han and Tibetan groups^a^.

Variable	Nanjing Han	Tibet Han	Tibetan	*p*^b^	*p*^c^	*p*^d^
**n**	151	80	278			
**Age, years**	34.87 ± 9.46	36.13 ± 11.72	36.30 ± 12.01	1.000	0.905	0.947
**Sex**				0.119^e^	0.664^e^	0.176^e^
Male	88	55	168			
Female	63	25	110			
**Hematological indices**						
**RBC (10**^12^**/L)**	4.83 ± 0.94	5.55 ± 0.66	5.02 ± 0.77	<0.001	0.010	<0.001
Male	5.11 ± 0.93	5.76 ± 0.54	5.14 ± 0.80	<0.001	0.979	<0.001
Female	4.44 ± 0.30	4.94 ± 0.59	4.81 ± 0.61	0.004	<0.001	0.766
**HGB (g/L)**	143.51 ± 15.40	175.95 ± 19.00	157.23 ± 23.34	<0.001	<0.001	<0.001
Male	153.52 ± 10.61	181.93 ± 14.82	163.26 ± 23.91	<0.001	<0.001	<0.001
Female	129.37 ± 8.43	158.30 ± 19.28	149.37 ± 19.81	<0.001	<0.001	0.192
**HCT (%)**	39.92 ± 6.96	53.10 ± 6.31	47.78 ± 7.26	<0.001	<0.001	<0.001
Male	41.90 ± 7.36	55.06 ± 5.22	49.33 ± 7.50	<0.001	<0.001	<0.001
Female	37.13 ± 5.25	47.30 ± 5.74	45.46 ± 6.25	<0.001	<0.001	0.200
**MCH (pg)**	29.75 ± 1.84	32.07 ± 3.41	31.75 ± 4.85	<0.001^f^	<0.001^f^	0.064^f^
**PLT (10**^9^**/L)**	224.32 ± 46.69	170.49 ± 45.24	182.53 ± 66.73	<0.001	<0.001	0.183

RBC, red blood cell counts; HGB, hemoglobin; HCT, hematocrit; MCH, mean corpuscular hemoglobin; PLT, blood platelet.

^a^The data are presented as the mean ± s.d.

^b^Tibet Han *vs* Nanjing Han.

^c^Tibetan *vs* Nanjing Han.

^d^Tibetan *vs* Tibet Han.

^e^Two-sided χ^2^ test.

^f^Mann-Whitney U-test.

**Table 3:** Spearman rank correlations between the validated increased plasma miRNAs and the hematological indices of the studied plasma samples (n = 509) (r/*P*).

Variable	RBC	HGB	HCT	PLT
miR-130a-3p	0.160	0.187	0.233	−0.092
	*P* = 0.0003	*P* < 0.0001	*P* < 0.0001	*P* = 0.0391
miR-302b-5p	0.135	0.180	0.252	−0.269
	*P* = 0.0023	*P* < 0.0001	*P* < 0.0001	*P* < 0.0001
miR-572	0.183	0.196	0.245	−0.184
	*P* < 0.0001	*P* < 0.0001	*P* < 0.0001	*P* < 0.0001
miR-629-5p	0.148	0.169	0.219	−0.178
	*P* = 0.0009	*P* = 0.0001	*P* < 0.0001	*P* < 0.0001

RBC, red blood cell counts; HGB, hemoglobin; HCT, hematocrit; PLT, blood platelet.

**Table 4:** Predicted target genes of the validated increased miRNAs in the Tibet Han population.

	Hypoxia-related			Erythroid-related			Megakaryocytic-related	
miRNA	target gene			target gene			target gene	
miR-130a-3p	DDX6	EGLN3	FOSL1	SMAD5	SP1		MAFB	MYB
	I7PR1	PXDN	PDE5A				HOXD1	PDGFRA
	TGFB2	TGFB1	UCP3				CXDCL2	MAFG
	CDKNN	SCAR3	TXNIP				CBFA	HOXA3
	CHRN2	PRKA1	EDN1					
miR-302b-5p	VEGFA	CREBP	EGLN1	SOX6	VEGFA	TGFB3	ETV6	RUNX1
	KCNM1	FOSL1	PPARA					
miR-629-5p	HIF3A	CD24	SLC8A1					
