# Supplementary material for: Influence of a high-altitude hypoxic environment on human plasma microRNA profiles
Source: Sci Rep. 2015 Oct 15;5:15156. doi: 10.1038/srep15156 (PMC4606833; doi:10.1038/srep15156)
Supplement: Supplementary Information [file srep15156-s1.doc]

**Influence of a high-altitude hypoxic environment on human plasma microRNA profiles**

Yan Yan^1,2,†^, Yonghui Shi^1,†^, Cheng Wang^1,2,†^, Pengtao Guo^3^, Junjun Wang^1^, Chen-Yu Zhang^2,*^ & Chunni Zhang^1,2, *^

^1^Department of Clinical Laboratory, Jinling Hospital, Nanjing University School of Medicine, State Key Laboratory of Analytical Chemistry for Life Science, Nanjing University, Nanjing 210002, China; ^2^Jiangsu Engineering Research Center for microRNA Biology and Biotechnology, Advance Research Institute of Life Sciences, Nanjing University, Nanjing, China; ^3^Department of Clinical Laboratory, the Forty- First Hospital of PLA, Nêdong, China.

**Address for correspondence:** Chunni Zhang, Department of Clinical Laboratory, Jinling Hospital, Nanjing University School of Medicine, Nanjing University, 305 East Zhongshan Road, Nanjing 210002, China. Phone: 86-25-80863082; Fax: 86-25-80861177. E-mail: [zchunni27@hotmail.com](mailto:zchunni27@hotmail.com). Chen-Yu Zhang, Advance Research Institute of Life Sciences, Nanjing University, 163 Xianlin Main Road, Nanjing 210046, China. E-mail: cyzhang@nju.edu.cn

**Keywords:** High-altitude-hypoxia, Tibet, Tibetan, Han Chinese, Plasma, MicroRNA

**Supplementary Material:**

**Supplementary Methods**

**The dynamic range of the spiking in exogenous miRNA**

We conducted an experiment to determine the optimal concentration of the exogenous miRNA needed to be added into plasma samples when performing RNA extraction. For this purpose, pooled plasma sample from 10 healthy persons was divided into ten identical portions (each 100 μL), the plasma was diluted with 300 μL DEPC water, and then 200 μL acid phenol was added, the mixture was vortexed vigorously and incubated at room temperature for 10 minutes. After the plasma proteins and RNAase were denatured completely, 20 μL exogenous miRNA, a plant miRNA MIR2911, with the concentrations of 0.1 fmol/L to 10^6^ fmol/L was added into the mixture, and then plasma RNA was extracted with the same following steps as we described in the Materials and Methods section in the manuscript. Finally, the RNA pellet was dissolved in 20 μL of RNase-free water and stored at -80°C until further analysis. The amounts of the exogenous miRNA which we added ranging from low to high levels in the ten samples were measured with a TaqMan probe-based qRT-PCR assay. The resulting Cq values were determined using fixed threshold settings and plotted versus the log_10_ of the amount of the exogenous miRNAs. Each sample and each concentration were run in triplicate for analysis. All data were collected and analyzed with an Applied Biosystems 7300 Sequence Detection System (Applied Biosystems, Foster City, CA, USA). The limit of detection and dynamic range for this exogenous miRNA was 1×10^2^ fmol/L and 1×10^2^ fmol/L～1×10^6^ fmol/L. According to the dynamic range of the miRNA, the concentration of 1×10^6^ fmol/L was chosen as the candidate one to be used as exogenous reference gene for plasma miRNA.

**Evaluation of repeatability of RNA extraction and RT-qPCR assay for exogenous reference gene**

The repeatability of RNA extraction and the analytical repeatability of qRT-PCR assay for this exogenous miRNA at 1×10^6^ fmol/L was assayed in order to confirm whether it was suitable as exogenous reference gene. The experiments were conducted as follows: Pooled plasma from 10 healthy persons was divided into ten identical portions (each 100 μL), and RNA was extracted from the ten pooled plasma samples using the same protocols as described above only except 20 μL of 1×10^6^ fmol/L exogenous miRNA was added into the mixture in each ten samples, respectively. And then the exogenous miRNA levels were measured with qRT-PCR assay. Each sample was run in triplicate for analysis. The data showed that the exogenous miRNA with the concentration of 1×10^6^ fmol/L added into plasma have a high repeatability and reproducibility.

**Supplementary Table S1.** Differently expressed miRNAs in pooled plasma samples from Tibet Han compared to that from Nanjing Han determined by TaqMan Low Density Assay^a^

| **miRNA** | **Nanjing Han**  **Cq** | **Tibet Han** | **Fold change**  **(Tibet Han/Nanjing Han)** |
| --- | --- | --- | --- |
|  |  | **Cq** |  |
| up-regulated |  |  |  |
| miR-646 | Undetermined | 14.042951 | 32433477.92 |
| miR-302b-3p | Undetermined | 22.081228 | 123199.8522 |
| miR-888-5p | Undetermined | 23.91004 | 34680.31783 |
| miR-661 | Undetermined | 24.95997 | 16774.25416 |
| miR-572 | Undetermined | 25.034348 | 15931.37867 |
| miR-22-3p | Undetermined | 26.049444 | 7871.511633 |
| miR-302b-5p | Undetermined | 26.869114 | 4466.150028 |
| let-7a-5p | Undetermined | 27.044086 | 3950.399928 |
| miR-501-5p | Undetermined | 28.009434 | 2023.217913 |
| miR-130a-3p | Undetermined | 28.931992 | 1067.394672 |
| miR-629-5p | Undetermined | 29.976665 | 517.4243003 |
| miR-943 | Undetermined | 30.008968 | 506.6908619 |
| miR-106b-3p | Undetermined | 30.026402 | 500.605038 |
| miR-326 | Undetermined | 30.89772 | 273.2635074 |
| miR-500a-5p | Undetermined | 30.947233 | 264.0444487 |
| miR-148b-3p | Undetermined | 30.976639 | 258.716992 |
| miR-511 | Undetermined | 30.983822 | 257.4319843 |
| miR-517a-3p | Undetermined | 31.032764 | 248.8452433 |
| miR-22-5p | Undetermined | 31.923988 | 134.358427 |
| miR-548d-5p | Undetermined | 31.962353 | 130.6458058 |
| miR-363-3p | Undetermined | 31.988468 | 128.3021023 |
| miR-323a-3p | Undetermined | 31.99525 | 127.7003793 |
| miR-1282 | Undetermined | 31.997478 | 127.6856866 |
| miR-598 | Undetermined | 31.99751 | 127.5004917 |
| miR-495 | Undetermined | 31.997612 | 127.4914776 |
| miR-638 | Undetermined | 32.003418 | 127.1610479 |
| miR-590-5p | Undetermined | 32.002884 | 127.0264392 |
| miR-199b-5p | Undetermined | 32.965443 | 65.18314244 |
| miR-410 | Undetermined | 32.975044 | 64.75075007 |
| miR-381 | Undetermined | 32.975063 | 64.74989733 |
| miR-1271-5p | Undetermined | 33.00476 | 63.52136458 |
| miR-101-3p | Undetermined | 33.955246 | 32.82272241 |
| miR-147a | Undetermined | 33.979618 | 32.2728929 |
| miR-26b-3p | 36.010307 | 29.997652 | 32.14646066 |
| miR-205-5p | Undetermined | 33.991405 | 32.01027132 |
| miR-132-3p | 32.019455 | 26.011564 | 31.99469925 |
| miR-337-5p | Undetermined | 33.996773 | 31.89143257 |
| miR-28-5p | 33.9762 | 27.98328 | 31.66442552 |
| miR-485-3p | Undetermined | 34.007668 | 31.65150072 |
| miR-183-5p | Undetermined | 34.00898 | 31.62272964 |
| miR-1254 | Undetermined | 34.06193 | 30.52668446 |
| miR-376c | 36.0054 | 30.98924 | 16.08933731 |
| miR-95 | 35.967888 | 30.956116 | 16.04045322 |
| miR-155-5p | 27.987488 | 23.932419 | 8.264568516 |
| miR-361-5p | 34.026093 | 29.984598 | 8.187165165 |
| miR-30a-5p | 23.978403 | 19.969732 | 8.01445157 |
| miR-30d-5p | 25.976606 | 21.96849 | 8.011366809 |
| miR-193b-3p | 25.977348 | 21.975077 | 7.967586076 |
| miR-660-5p | 29.980316 | 25.979797 | 7.9579123 |
| miR-140-5p | 30.979855 | 26.985304 | 7.925058034 |
| miR-140-3p | 32.008835 | 28.014797 | 7.922237762 |
| miR-29c-3p | 31.985264 | 27.991554 | 7.920436828 |
| miR-26a-1-3p | 35.975956 | 31.986258 | 7.909743987 |
| miR-339-3p | 28.971733 | 24.988005 | 7.865835417 |
| miR-519a-3p | 36.064396 | 32.96949 | 4.247977133 |
| miR-24-3p | 23.002056 | 19.95698 | 4.103762388 |
| miR-222-3p | 22.982443 | 19.942234 | 4.08994006 |
| miR-1290 | 24.012028 | 20.976389 | 4.082835382 |
| miR-181a-2-3 | 32.985146 | 29.953707 | 4.070965514 |
| miR-30c-5p | 26.984592 | 23.953218 | 4.064970831 |
| miR-29b-3p | 29.964346 | 26.939613 | 4.046301997 |
| miR-19b-3p | 25.985703 | 22.964685 | 4.035893207 |
| miR-454-3p | 29.974077 | 26.954185 | 4.032744487 |
| miR-200a-3p | 29.993675 | 26.97523 | 4.028707321 |
| miR-20b-5p | 31.000763 | 27.98407 | 4.023815064 |
| miR-196b-5p | 32.982517 | 29.972803 | 4.004394192 |
| miR-16-5p | 23.989681 | 20.980642 | 4.002523297 |
| miR-126-3p | 24.981888 | 21.974577 | 3.997731834 |
| miR-19a-3p | 31.986212 | 28.982605 | 3.987481982 |
| miR-146b-5p | 27.970434 | 24.96982 | 3.979218169 |
| miR-223-5p | 29.982813 | 26.98646 | 3.973154789 |
| miR-942 | 27.97307 | 24.977415 | 3.971232975 |
| miR-342-5p | 31.044464 | 28.048811 | 3.965561067 |
| miR-345-5p | 27.976427 | 24.980923 | 3.965151529 |
| miR-186-5p | 25.977427 | 22.982485 | 3.96360309 |
| miR-1180 | 30.001095 | 27.015907 | 3.942529363 |
| miR-151a-3p | 25.976213 | 22.994768 | 3.932312563 |
| miR-93-5p | 27.953318 | 24.97015 | 3.931385944 |
| miR-221-3p | 29.992903 | 27.01414 | 3.919405931 |
| miR-223-3p | 20.957151 | 17.98047 | 3.913750262 |
| miR-182-5p | 36.98426 | 34.01433 | 3.895477009 |
| miR-652-3p | 29.94881 | 26.99882 | 3.842011933 |
| miR-517c-3p | 35.936646 | 33.01543 | 3.766138353 |
| miR-335-5p | 35.948425 | 33.0436 | 3.723599735 |
| miR-18a-5p | 36.059704 | 33.93302 | 2.171290863 |
| miR-99a-3p | 36.035538 | 33.977383 | 2.07352724 |
| miR-629-3p | 31.994308 | 29.949663 | 2.05420192 |
| miR-141-3p | 32.00067 | 29.968578 | 2.033498603 |
| miR-125a-5p | 26.999773 | 24.970486 | 2.029548758 |
| miR-99b-3p | 33.01845 | 30.992678 | 2.027505742 |
| miR-202-3p | 34.02686 | 32.00057 | 2.025337027 |
| let-7g-5p | 27.985725 | 25.959578 | 2.025136285 |
| miR-532-3p | 26.976702 | 24.950668 | 2.024974864 |
| miR-451a | 26.985605 | 24.960985 | 2.02299254 |
| miR-144-5p | 35.995888 | 33.97625 | 2.018900765 |
| miR-142-3p | 29.964397 | 27.942993 | 2.018487986 |
| miR-212-3p | 33.030243 | 31.012459 | 2.013429559 |
| miR-29a-3p | 29.974474 | 27.956816 | 2.013254419 |
| miR-93-3p | 27.988575 | 25.973246 | 2.012881162 |
| miR-30b-5p | 26.987345 | 24.97201 | 2.010014627 |
| miR-210 | 26.973562 | 24.967218 | 2.007526995 |
| miR-374b-5p | 31.005762 | 28.994295 | 2.004630029 |
| miR-532-5p | 29.01202 | 27.000828 | 2.00425212 |
| miR-21-5p | 27.993462 | 25.98301 | 2.003222956 |
| miR-885-5p | 27.969809 | 25.960258 | 2.001970895 |
| down-regulated | |  |  |
| miR-628-5p | 13.921893 | 36.023155 | 1.10505E-07 |
| miR-523-3p | 20.96803 | 34.98392 | 3.00137E-05 |
| miR-190b | 19.036366 | 33.0283 | 3.05599E-05 |
| miR-520d-3p | 20.026468 | 34.017426 | 3.05806E-05 |
| miR-23a-3p | 28.037054 | Undetermined | 0.000124542 |
| miR-130b-3p | 28.881708 | Undetermined | 0.000223657 |
| miR-296-3p | 30.96416 | Undetermined | 0.000947249 |
| miR-1303 | 30.969934 | Undetermined | 0.000952407 |
| miR-605 | 30.977947 | Undetermined | 0.000957712 |
| miR-124-3p | 31.967209 | Undetermined | 0.001898504 |
| miR-503 | 31.989899 | Undetermined | 0.001928599 |
| miR-512-3p | 31.990393 | Undetermined | 0.001929261 |
| miR-219-5p | 32.015873 | Undetermined | 0.001963636 |
| miR-576-3p | 32.957832 | Undetermined | 0.003772412 |
| miR-522-3p | 32.9671 | Undetermined | 0.003796719 |
| miR-518e-3p | 33.98882 | Undetermined | 0.007708629 |
| miR-516a-3p | 33.990498 | Undetermined | 0.007728638 |
| miR-15a-3p | 34.03384 | Undetermined | 0.007964354 |
| miR-1244 | 28.97593 | 32.992603 | 0.030761273 |
| miR-328 | 24.992224 | 28.011265 | 0.061333977 |
| miR-192-3p | 31.961683 | 34.97597 | 0.061624421 |
| miR-802 | 32.976837 | 35.971703 | 0.062459594 |
| miR-484 | 19.989376 | 22.975515 | 0.062748797 |
| miR-518f-3p | 18.956673 | 21.030197 | 0.118121722 |
| miR-31-3p | 29.9849 | 32.003323 | 0.122896012 |
| miR-1274a(16) | 20.992771 | 22.996609 | 0.124144762 |
| miR-26b-5p | 29.992033 | 31.991785 | 0.124318939 |
| miR-302a-3p | 18.021122 | 20.018274 | 0.124543229 |
| miR-145-3p | 33.98727 | 35.98148 | 0.124976002 |
| miR-489 | 33.983536 | 35.97123 | 0.125362434 |
| miR-7-5p | 32.01566 | 34.003727 | 0.125509285 |
| miR-483-5p | 21.961416 | 23.945082 | 0.125712978 |
| miR-548c-3p | 33.97121 | 35.953136 | 0.125864645 |
| miR-224-5p | 30.973103 | 32.952595 | 0.126077173 |
| miR-197-3p | 24.996 | 26.971981 | 0.126384373 |
| miR-720 | 16.997301 | 18.972656 | 0.126620034 |
| miR-27a-3p | 29.081205 | 30.9381 | 0.137259461 |
| miR-34a-3p | 30.828539 | 32.030186 | 0.216476902 |
| miR-411-5p | 32.935394 | 33.993122 | 0.238844407 |
| miR-372 | 30.985817 | 32.021656 | 0.242495688 |
| miR-618 | 19.007439 | 20.036732 | 0.243598609 |
| miR-181a-3p | 34.973503 | 36.00085 | 0.244276164 |
| miR-1274b | 14.970381 | 15.971247 | 0.248801453 |
| miR-185-5p | 26.97916 | 27.97237 | 0.249768077 |
| miR-642a-5p | 29.992214 | 30.985064 | 0.24983041 |
| miR-708-5p | 31.020187 | 31.974596 | 0.25657651 |
| miR-296-5p | 29.012604 | 29.955608 | 0.258612874 |
| miR-30d-3p | 33.04377 | 33.98062 | 0.260089849 |
| miR-19b-1-5p | 9.07875 | 9.967512 | 0.268905282 |
| miR-339-5p | 25.920609 | 25.998089 | 0.471193323 |
| miR-181a-5p | 29.9754 | 30.02313 | 0.481011403 |
| miR-25-5p | 28.964075 | 28.99983 | 0.485713335 |
| miR-122-5p | 21.973295 | 22.004662 | 0.486497636 |
| miR-1255b-5p | 29.98129 | 30.0088 | 0.488497472 |
| miR-543 | 32.99077 | 33.00888 | 0.491689687 |
| miR-518b | 33.006718 | 33.0226 | 0.491747628 |
| miR-375 | 24.959698 | 24.975288 | 0.491846554 |
| miR-422a | 31.979279 | 31.992231 | 0.492747002 |
| miR-30e-3p | 29.99142 | 30.004362 | 0.493455195 |
| miR-1260a | 23.992857 | 24.005175 | 0.493668569 |
| miR-31-5p | 27.985847 | 27.99248 | 0.494909966 |
| miR-886-3p | 26.964552 | 26.970976 | 0.494981496 |
| miR-127-3p | 28.9692 | 28.975489 | 0.495027988 |
| miR-486-5p | 21.975529 | 21.97846 | 0.496181207 |
| miR-218-5p | 30.975052 | 30.977888 | 0.496214225 |
| let-7c | 28.975546 | 28.976791 | 0.496761751 |
| miR-1300 | 30.977125 | 30.979246 | 0.497170638 |

^a^ "undetermined" is referred as the Cq value of 40 when the fold change is calculated.

**Supplementary Table S2.** Relative miRNA concentrations to MIR2911 in the plasma

samples from Nanjing Han, Tibet Han and Tibetan determined by individual qRT- PCR assay^a^

| **miRNA** | **Nanjing Han**  **(n = 151)** | **Tibet**  **Han**  **(n = 80)** | **Fold**  **change^b^** | ***P*^c^** | **Tibetan**  **(n = 278)** | **Fold**  **change^d^** | ***P*^e^** | ***P*^f^** |
| --- | --- | --- | --- | --- | --- | --- | --- | --- |
| miR-130a-3p | 239±9.57 | 529±54.08 | 2.21 | < 0.0001 | 488±27.43 | 2.04 | < 0.0001 | 0.710 |
| miR-302b-5p | 4.33±0.13 | 13.22±1.49 | 3.05 | < 0.0001 | 9.89±0.50 | 2.28 | < 0.0001 | 0.016 |
| miR-572 | 16.25±0.64 | 32.61±3.15 | 2.01 | < 0.0001 | 23.99±0.93 | 1.48 | < 0.0001 | 0.082 |
| miR-629-5p | 7.17±0.31 | 18.32±2.61 | 2.56 | < 0.0001 | 14.88±0.86 | 2.08 | < 0.0001 | 0.908 |

^a^miRNA data are presented as the mean ± SEM (×10^-5^).

^b^Tibet Han/Nanjing Han.

^c^Tibet Han *vs* Nanjing Han.

^d^Tibetan/Nanjing Han.

^e^Tibetan *vs* Nanjing Han.

^f^Tibet Han *vs* Tibetan.

**Supplementary Table S3**. The plasma concentrations of VEGFA and EPO in Nanjing Han, Tibet Han and Tibetan^a^

| **Variable** | **Nanjing Han** | **Tibet Han** | **Tibetan** | ***p*^b^** | ***p^c^*** | ***p^d^*** |
| --- | --- | --- | --- | --- | --- | --- |
| **n** | 64 | 46 | 64 |  |  |  |
| **Age, years** | 34.65±8.70 | 36.51±9.87 | 35.42±9.76 | 0.891 | 0.986 | 0.983 |
| **Sex** |  |  |  | 0.977^e^ | 0.723^e^ | 0.724^e^ |
| **Male** | 36 | 26 | 34 |  |  |  |
| **Female** | 28 | 20 | 30 |  |  |  |
| **VEGFA (pg/ml)** | 89.24±3.76 | 116.34±8.34 | 118.98±8.03 | 0.012^f^ | 0.001^f^ | 0.99^f^ |
| **EPO (mU/ml)** | 109.51±10.50 | 165.48±13.13 | 168.48±15.03 | 0.0002^f^ | 0.0008^f^ | 0.45^f^ |

^a^Age is presented as mean ± s.d and other data are presented as the mean ± SEM.

^b^Tibet Han *vs* Nanjing Han.
^c^Tibetan *vs* Nanjing Han.
^d^Tibetan *vs* Tibet Han.
^e^Two-sided χ^2^ test.
^f^Mann-Whitney U-test.

**Figure S1**

**
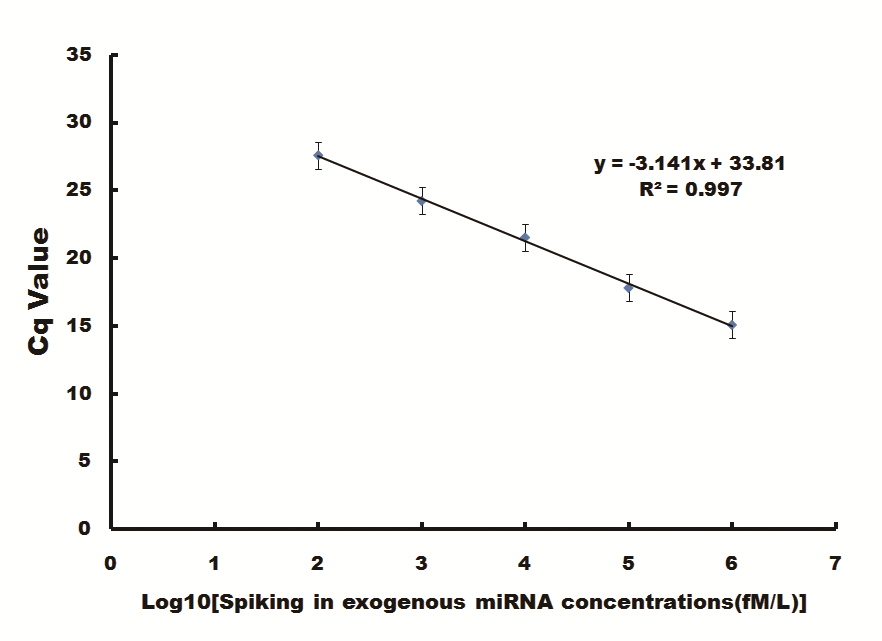
**

**Supplementary Figure S1. The dynamic range of the spiking in exogenous miRNA MIR2911.**

**Figure S2**

**
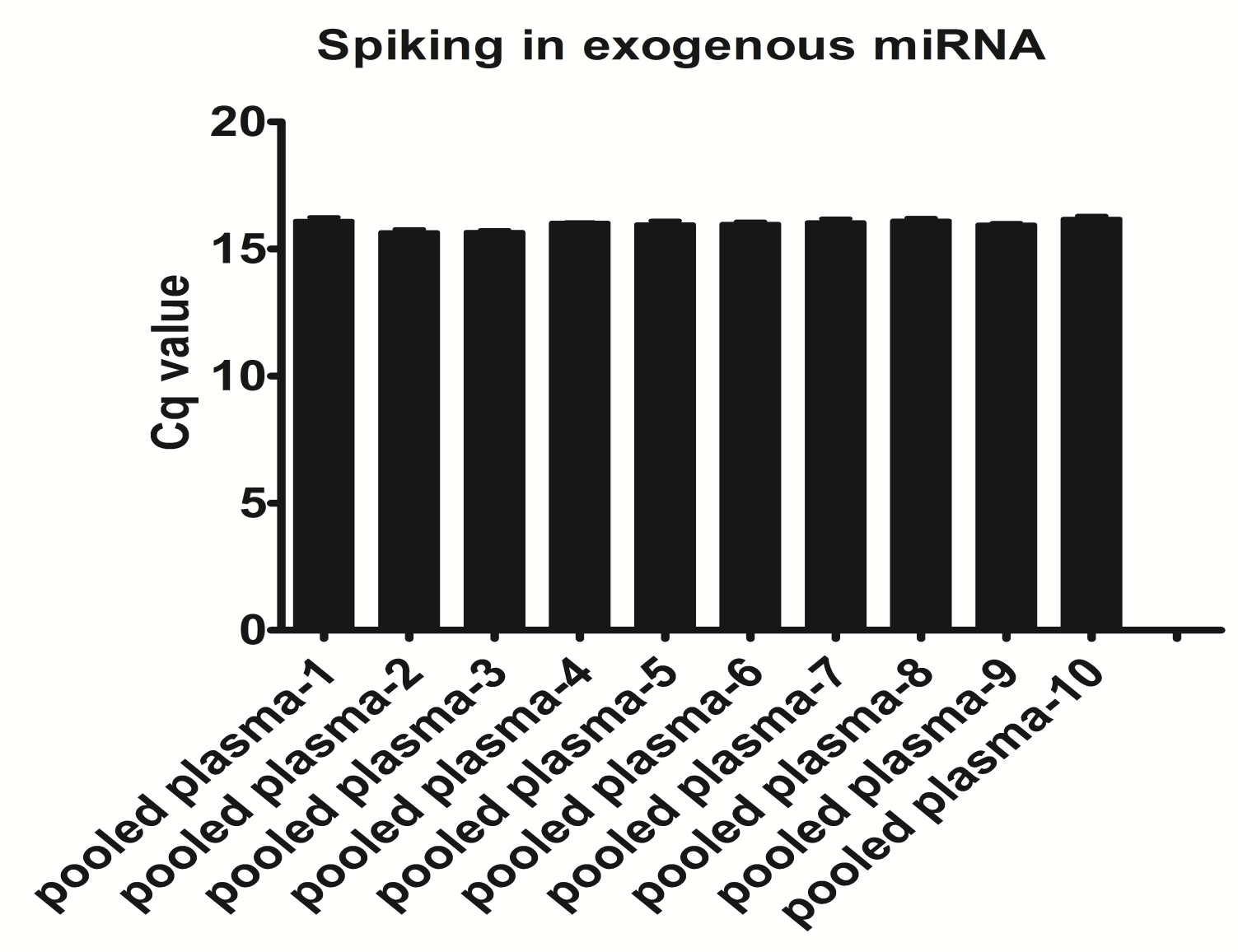
**

**Supplementary Figure S2. The Cq values of the ten pooled plasma sample spiked in exogenous miRNA MIR2911 with the concentration of 1×10^6^ fmol/L.** The data are presented as means ± SEM.

**Figure S3**

**Supplementary Figure S3. The levels of increased plasma miRNAs in peripheral blood cells from the Nanjing Han (n = 30), Tibet Han (n = 30) and Tibetan (n = 30) groups.**

**Figure S4**

**Supplementary Figure S4.** **Spearman rank correlations between the upregulated 4 miRNAs in all individuals (n = 509).**

**Figure S5**

**Supplementary Figure S5.** **Spearman rank correlations between the validated upregulated miRNAs in Tibet Han and RBC (n = 509).**

**Figure S6**

**Supplementary Figure S6. Spearman rank correlations between the validated upregulated miRNAs in Tibet Han and HGB (n = 509).**

**Figure S7**

**Supplementary Figure S7. Spearman rank correlations between the validated upregulated miRNAs in Tibet Han and HCT (n = 509).**

**Figure S8**

**Supplementary Figure S8. Spearman rank correlations between the concentrations of VEGFA and validated 4 increased miRNAs in the plasma samples from Nanjing Han, Tibet Han and Tibetan (n = 174).**

**Figure S9**

**Supplementary Figure S9. Spearman rank correlations between the concentrations of EPO and validated 4 increased miRNAs in plasma samples from Nanjing Han, Tibet Han and Tibetan (n = 174).**
